# Adalimumab in Pediatric Inflammatory Bowel Disease

**DOI:** 10.3389/fped.2022.852580

**Published:** 2022-04-13

**Authors:** So Yoon Choi, Ben Kang

**Affiliations:** ^1^Department of Pediatrics, Kosin University Gospel Hospital, Kosin University College of Medicine, Busan, South Korea; ^2^Department of Pediatrics, School of Medicine, Kyungpook National University, Daegu, South Korea

**Keywords:** adalimumab, biologics, inflammatory bowel disease, Crohn's disease, ulcerative colitis, child

## Abstract

The introduction of biological agents with strong anti-inflammatory action, such as antitumor necrosis factor (TNF) agents, has changed inflammatory bowel disease (IBD) treatment strategy and goals, and has contributed significantly to improve the long-term prognosis of patients. Moreover, several biological agents are being used or researched in pediatric populations. However, only two biological agents, infliximab (IFX) and adalimumab (ADL), are currently approved for children and adolescents. In pediatric IBD, there are limitations and burdens associated with facilitating mucosal healing (MH) when utilizing these two biological agents. ADL is effective in both naïve patients and those with previous experience with biologics. Beyond clinical remission, this drug is also effective for MH and histological remission. The use of therapeutic drug monitoring to further enhance the effectiveness of ADL treatment can be expected to reduce treatment failure of ADL and pave the way for appropriate treatment in the treat-to-target era. This review paper focuses on ADL, examine studies conducted in children, and determine the role this agent plays against pediatric inflammatory bowel disease.

## Introduction

Treatment strategies and goals for inflammatory bowel disease (IBD) have undergone a series of changes over time and are still in the process of development. For so long, various symptom-based treatment criteria have been established for the effective treatment of IBD (1). Assessing patients based on symptoms has been the easiest and most intuitive method (2, 3). Even in children, the Pediatric Crohn's Disease Activity Index (PCDAI) and Pediatric Ulcerative Colitis Activity Index had mainly been used as indices that converted the sum of clinical indicators into scores, along with symptoms, such as diarrhea, abdominal pain, and general well-being, which are still commonly used today (4, 5).

However, while IBD treatment aiming at clinical remission could reduce the rate of intestinal damage, current literature do not include data supporting change of the disease course (3). Given that many cases have led to surgeries following complications that occurred due to disease progression despite treatment, it has become evident that the traditional treatment strategy of targeting mere symptom resolution is problematic (6, 7). Hence a new treatment goal of mucosal healing (MH) has consequently emerged. Several studies have revealed that MH is a predictor of a good, long-term clinical outcome, such as a reduction in recurrence, hospitalization rate, and rate of surgery (8–11). Therefore, endoscopic remission has been regarded as the most important goal in the IBD treatment strategy. Despite the lack of evidence, a better long-term prognosis can be expected provided that histological remission is added to the outcomes (12).

The introduction of biological agents with strong anti-inflammatory action, such as anti-tumor necrosis factor (TNF) agents, have greatly contributed to changes in IBD treatment strategy and goals, which is advantageous for the long-term prognosis of patients (13–15). Moreover, several biological agents are being used or researched in pediatric populations (16, 17). However, only two biological agents, namely infliximab (IFX) and adalimumab (ADL), have currently been approved for children and adolescents (18). In pediatric IBD, there are limitations and burdens associated with facilitating MH utilizing both biological agents.

Since IFX had first been used in pediatric IBD, numerous studies on the same have emerged. Although its use for pediatric ulcerative colitis (UC) is still limited in some countries, it has been widely used since ADL was approved for pediatric Crohn's disease (CD). However, only a limited number of pediatric studies regarding ADL have currently been available. This review paper will therefore focus on ADL, examine the studies conducted in children, and determine the role of this agent in pediatric IBD.

## ADL And Its Indication for Pediatric IBD

ADL is a 100% human monoclonal anti-TNF antibody that binds specifically to TNF alpha (19). This antibody regulates the inflammatory response by blocking the signal transduction process that causes the inflammatory response by binding to the TNF receptor present in the cell membrane. Children under and over 40 kg generally receive a 20 and 40 mg subcutaneous injection every 2 weeks, respectively, after the initial induction treatment (20). ADL can be used for the treatment of moderately to severely active CD in pediatric patients (from 6 years of age) who have had an inadequate response to conventional therapy including primary nutrition therapy and a corticosteroid and/or an immunomodulator, or who are intolerant to or have contraindications for such therapies.

ADL had first been approved for pediatric CD in 2012 and has since been used for more than 9 years, during which a substantial amount deal of empirical data had been accumulated.

## Efficacy of ADL in Pediatric IBD

### ADL for Clinical Remission

The first studies on the effects of ADL in children can be traced backed to 2008. Notably, a 2008 retrospective study of 10 patients with both CD and UC in the United States showed that clinical remission rate at 24 weeks was quite high at 80% (21). Another study published within the same year reported that ~50% of patients had discontinued steroids for 3 months or more at 26 weeks (22). Both of these studies included patients who had failed IFX treatment. A 2009 study published in Italy that examined 9 out of 23 naïve patients found that a clinical remission rate of 65.2% at 24 weeks (23). However, most of the published studies had limitations in that they were conducted retrospectively and included patients who did not respond to or whose response vanished after receiving IFX treatment. Moreover, short-term clinical remission rates from 2 weeks to up to 2 years were reported (Table 1).

**Table 1:** Efficacy for clinical remission of adalimumab.

**References**	**Design**	**Number**	**Outcome**	**Time point**	**Remission rate %**	**Serious adverse reaction**
Noe et al. (21) USA	Retrospective	10 (7 CD,3 UC)	PCDAI <10	W 24	80	(-)
Wyneski et al. (22) USA	Retrospective	14 (All previous IFX)	Steroid-free interval >3 months	W 26	50	(-)
Viola et al. (23) Italy	Retrospective	23 (9 naïve, 14	PCDAI ≤ 10	W 2	36.3	(-)
		unresponsive to IFX)		W 4	60.8	
				W 12	30.5	
				W 24	65.2	
Rosh et al. (24) USA	Retrospective	115 (IFX experienced	PGA	W 12	65	(-)
		95%)		W 24	71	
				W 48	70	
Rosenbach et al. (25) Israel	Retrospective	14 (71% previous IFX)	Harvey-Bradshaw score <4	W 69	50	1/14(7%)
Russell et al. (26) UK	Retrospective	72 (IFX experienced 94%)	PDCAI ≤ 10	W 4	24	4/72(6%)
				W 24	58	
				W 48	41	
Nobile et al. (27) Italy	Retrospective	19 (79% previous IFX)	PGA standard definition	W 114	23.1	(-)
Fumery et al. (28) France	Retrospective	27(IFX failure)	PGA=1	W 64	70	(-)
Cozijnsen et al. (29)	Prospective	53 (IFX refractory CD)	PCDAI ≤ 12.5	W 4	21	1/53(2%)
Netherlands				W 16	38	
				W 32	57	
				W 52	53	
				W 104	47	
Navas-López et al. (30)	Retrospective	62(All naïve)	wPCDAI < 12.5	W 12	80.6	Adverse effects
Spain				W 52	95	8(13%)
Alvisi et al. (31) Italy	Retrospective	44	PCDAI ≤ 10	W 24	55	2/44(4.5%)
				W 48	78	
				W 72	52	

*IFX, infliximab; CD, Crohn's disease; PGA, Physical Global Assessment; PCDAI, Pediatric Crohn's Disease Activity Index; CDAI, Crohn's Disease Activity Index; wPCDAI, The weighted Pediatric Crohn's disease activity index*.

A study published in 2012 also contributed to the approval of ADL in children and was the first prospective RCT study in the treatment of pediatric ADL (20). After the introductory treatment, the clinical effect and safety were evaluated by dividing the participants into a high and low-dose group. Among the participants in the high-dose group, those who weighed ≥40 kg and <40 kg received 40 and 20 mg of ADL every 2 weeks, respectively. Among the participants in the low-dose group, those who weighed ≥40 kg and <40 kg received 20 and 10 mg of ADL every 2 weeks. In conclusion, the high-dose group showed better effects, with no differences in safety between the two groups. Thus, the higher dose study was selected as a useful guide in the treatment of children. When treated with a high dose, the clinical remission rate was 38.7% at 26 weeks and 33.3% at 52 weeks. Moreover, among the children who received a high dose, naïve patients had significantly higher clinical remission rates at 26 weeks than IFX-experienced patients (56.9 vs. 16.7%). At 52 weeks, the clinical remission rate was still higher in the naïve group. In addition, 38 patients (40.0%) in the low-dose group and 33 patients (35.5%) in the high-dose group received steroids at the start of the study. Among these patients, 65.8% (25/38) and 84.8% (28/33) discontinued steroid use at week 26, whereas 26.3% (10/38) and 33.3% (11/33) had achieved clinical remission at week 26 without steroid use, respectively.

Among the pediatric studies on ADL published after its approval in Europe in 2012, a prospective study published in the Netherlands in 2015 included 53 CD patients with non-response or secondary loss of response to IFX (29). A PCDAI score of 12.5 or less was considered to be an outcome, and the clinical remission rate after 2 years of treatment was reported to be 47%. In a 2018 retrospective study in Spain, ADL treatment results in 62 naïve patients showed a clinical remission rate of 80.6 and 95% at 12 and 52 weeks, respectively. The personalized anti TNF therapy in CD study (PANTS) of children 6 years of age and older and adults, reported clinical response of 13.1% and a clinical remission of 42.3% at 14 weeks of ADL treatment (32). Although this study had a limitation that only 18% were under the age of 18, it is significance that it is as a large-scale prospective observational study. In conclusion, the efficacy of adalimumab in clinical remission in pediatric patients continues to be demonstrated.

### ADL for Fistula Remission

In the IMAgINE study, 21 and 15 patients in the low- and high-dose groups had one or more fistulas, respectively. By week 52, 23.8% (5/21) of the patients in the low-dose group achieved fistula remission, whereas 28.6% (6/21) had a reduction in the number of fistulas (20). Fistula remission was also achieved in 40% (6/15) of patients in the high-dose group. In follow-up studies, fistula closure and improvement rates were sustained until 280 weeks (33). This suggests that ADL has long-term effects on fistula CD.

### ADL for Growth Improvement

ADL treatment was also effective in the recovery of patients' growth in height. At the start of IMAgINE 1, the median height velocity z score was −2.81, but at the start of the IMAgINE 2 study, the median height velocity z score recovered to 1.7, and was maintained well until week 192 of the treatment (34). In another paper, it was found that growth rate recovery was statistically significantly at Week 52 of ADL treatment in patients whose height growth rate had decreased at the start of treatment (35). A recent study reported by Matar et al. showed that ADL treatment resulted in a significant improvement in linear growth and normalization for weight and body mass index at 72 weeks of treatment in children with moderate to severe CD (36).

### ADL for Endoscopic and Histopathological MH

Most pediatric studies evaluating the effects of ADL have focused primarily on clinical remission or duration of steroid discontinuation. Very few pediatric studies have been available regarding the effects of ADL treatment on MH, which is considered an important IBD treatment goal. In 2014, the first study on the MH effect of ADL treatment in children had been published in Italy (27). Although this was a retrospective study that mostly included patients who had previously used IFX, the endoscopic remission rate was reported to be 25% at week 154 of ADL treatment. Even after comparison with IFX, the MH rate was not inferior. Since then, a 2017 study evaluating histological remission and MH associated with ADL in the treatment of pediatric CD had been published (37). This was a retrospective study including approximately 30% of patients who had experience with IFX. After induction treatment, 17.4% of patients had MH, and PCDAI and simple endoscopic score for CD (SES-CD) were significantly decreased. No significant difference in the rate of MH, including histological remission, was observed before and after treatment. A 2020 prospective study published in South Korea on 17 children with moderate to severe CD who had no experience with biologics assessed the therapeutic effects on clinical remission, MH, and histological remission at 4 months after the initiation of ADL (38). At 4 months of treatment, clinical remission was achieved in 14 of the 17 patients (82.4%), MH was achieved in 47.1%, and histological remission was achieved in 23.5%.

This suggests that ADL may play a role in achieving MH as well as in histological remission among children. However, there are possible limitations in the aforementioned results given the small number of subjects and potential for selection bias.

### Efficacy for Pediatric UC Patients

Although ADL has been used in adult UC, with published studies confirming its effectiveness, a few studies have investigated the effects of ADL on pediatric UC given that ADL has not yet been approved for the treatment of pediatric UC.

In 2015, a small study was published in the UK on 11 children with refractory UC who had previous experience with IFX treatment (39). The results showed that 6 of 11 children exhibited a reduction in PUCAI to <10 points after 6 months of treatment. Moreover, in a 2018 study involving 32 patients with IFX experience published in JPGN, clinical remission was achieved in 53% of patients at 12 weeks, 47% at 30 weeks, and 41% at 52 weeks (40). MH, which was also evaluated at week 52, was achieved in 28%. Among patients who underwent endoscopy, a Mayo score of 0 was defined as MH, whereas in those without endoscopic assessment, a fecal calprotectin of <250 μg/g was considered to be a surrogate marker for MH. It was therefore difficult to view this as a complete assessment. Nonetheless, the aforementioned study offers further confirmation that ADL is effective in pediatric UC. The largest phase 3 study of ADL treatment supported that ADL is an efficacious and safe treatment option for children with moderate to severe UC (41).

### Long-Term Efficacy of ADL

Pediatric studies on the long-term effects of ADL treatment are scarce. A follow-up study of the IMAgINE 1 study in 2017 demonstrated the long-term efficacy of ADL after 5 years of treatment, with a clinical remission rate of 41% and a clinical response rate of 48% at 5 years of treatment (34). A paper published in 2021 reported on patients who received a 3-year course of ADL for pediatric CD (42). ADL trough levels (TLs) were significantly higher in patients who achieved clinical or endoscopic remission during the first year of treatment, with the effect being sustained in the long-term, and a positive effect on growth was shown. This demonstrates that ADL is also effective in maintaining long-term remission. A recent study reporting the outcomes of long-term treatment with ADA also showed that ADA monotherapy maintained sustained clinical remission for more than 1 year, and nearly 80% of the anti-TNF naïve patients continued to receive ADA therapy during follow-up throughout pediatric care (43). However, this study was conducted retrospectively, and the median follow-up on ADA under pediatric care was only 24.8 (interquartile range 15.6–38.4) months. More well-designed studies on long-term effects can be expected to be published in the future.

### Failure and Loss of Response to ADL

Although treatment with ADL has been able to promote clinical remission, MH, and even histological remission, a number of patients still do not respond to ADL or have secondary loss of response (LOR). Although the clinical remission rate has remained at ~50% in the first year of treatment, evidence has suggested that the ADL treatment failure rate also increases to ~24% in the first year and 42% in the second year (29). In addition, the effects of ADL had been found to be lower in patients who were initially non-responders to IFX, as well as those who had an initial response to prior IFX treatment and subsequently LOR. Another study reported that the treatment failure rate for ADL was up to 54.9% at year 1 (28). However, these studies had limitations as they were not IFX naive patients, but patients who had previously failed or did not respond to IFX treatment. The PANTS study which included anti TNF naïve patients reported that the rate of primary non-responder was 26.8% (32). The study reported an association with low ADL TL mediated by immunogenicity as a predictive factor for treatment failure. The main mechanism causing anti TNF treatment failure and LOR is immunogenicity due to the formation of anti-drug antibodies (32). These antibodies prevent anti-TNF alpha agents from binding to the receptor or promote drug clearance through the reticuloendothelial system. The formation of antibodies to TNF alpha antagonists correlates with lower serum drug levels and shorter duration of responses (44). LOR may also be associated with individual differences in bioavailability and pharmacokinetics leading to immunogenicity or other factors that increase drug clearance (45).

## Safety and Adverse Events During ADL Therapy

Around half of the patients have reported adverse events of ADL, with the most common being infection, followed by injection site adverse reactions, joint pain, and muscle pain (46). Serious adverse events occur in 11.5% of patients, among which hematological side effects are the most common. Reports have shown that 35.2% of cases required drug discontinuation due to these adverse reactions. Dulai et al. found that the risk of lymphoma in children treated with anti TNF therapy was not greater than with other therapies, and was significantly lower with respect to serious infections (47). In another retrospective observational study, the majority of delayed adverse events were infectious diseases, and most of them were mild upper respiratory tract infections (48). Among non-infectious causes, paradoxical psoriasis accounted for the largest proportion, and its incidence was higher in the ADL group than in the IFX group. Previous studies have reported a higher incidence of these dermatological adverse events in ADL treatment compared with IFX, but the reasons are still unclear (49, 50). More studies evaluating the safety and adverse effects of ADL treatment in children are still needed.

## Maximizing the Efficacy While Minimizing the Side Effects of ADL in Pediatric IBD

### Effects of Concomitant Immunomodulators

Studies showing the ability of the combined use of anti-TNF agents and IMMs in reducing immunogenicity and increasing effectiveness have mainly been conducted on adults (47, 51). Although there are concerns about side effects such as opportunistic infection and hepatosplenic T-cell lymphoma in children, the effectiveness of the combination therapy of IFX and IMM has been demonstrated to some extent (52).

Nonetheless, controversy regarding the effectiveness of ADL and IMM combination therapy and ADL monotherapy still remains. A retrospective study on children showed that the 1-year remission rate was significantly higher with IMM and combined treatment than with ADL alone (26). However, the post-mortem analysis of the IMAgINE 1 study showed different results. Accordingly, no significant difference in treatment response and clinical remission rates at 4, 26, and 52 weeks were observed between the ADL and IMM combination therapy group and the ADL alone group (20). Moreover, no significant difference in ADL drug concentrations was noted between the two groups at weeks 4, 26, and 52. A current study found no significant difference in the ADL TLs between patients treated with azathioprine and ADL and those treated with ADL monotherapy (38). Another recent study comparing combination vs. monotherapy at 72 weeks of treatment found no significant difference in the rates of sustained corticosteroid-free clinical remission or sustained composite outcome of clinical remission. These results supported that combination therapy of ADL and IMM was not more effective than ADL monotherapy in pediatric CD (53). In the recent PANTS cohort study, the proportion of ADL-treated patients not in remission at week 54 did not differ between patients receiving combination therapy with IMM and patients receiving monotherapy (32). The ECCO-ESPGHAN guidelines also stated that ADL monotherapy would be an alternative to combination therapy of ADL and IMM in patients naïve to anti-TNF agents (54).

A study on adults showed that the remission rate of ADL and IMM combination therapy was slightly higher than monotherapy immediately after introductory treatment, but the remission rate or dose escalation at 1 year was similar in both groups (55).

Though combination treatment may have some effects on the remission rate at the time of the initial introduction of treatment, there may not be many benefits in the long-term effect, suggesting the need for carful interpretation of these results.

### Therapeutic Drug Monitoring of ADL

A subanalysis of the IMAgINE 1 study on ADL TLs measured at weeks 4, 8, 16, 26, and 52 showed that compared with the low-dose group, the high-dose group (i.e., ≥40 and 20 mg administered to those weighing ≥40 and <40 kg every other week) had a higher average ADL TL (20). In addition, the mentioned study found that cases of dose escalation from biweekly to weekly were much higher and that the ADL TL also increased with the dosage.

A comparison of the clinical remission rate by dividing the ADL TL into two groups based on the median value showed that the group with an ADL TL higher than the median value at the fourth week of treatment had a significantly higher clinical remission rate (44). This suggests that those with higher ADL TL may have higher remission rates. In this study, anti-ADL antibody (AAA) at week 52 was observed in 6 out of 182 patients (3.3%). Overall, the ADL TL was lower in AAA-positive patients. Relevant factors that increase the clearance of ADL, that is, decrease TL, have been identified, and clearance rates appear to be higher in patients with prior IFX, those with antibody-generated IFX, and those not using IMM. Nevertheless, there is significant overlap between the two groups despite no significant difference between them. Furthermore, at the beginning of the initial treatment, the higher weight, higher CRP, lower albumin, and higher PCDAI score seemed to indicate a higher clearance rate; however, this also showed overlap with no significant difference.

A South Korean pediatric study comparing the ADL TL between patients show did and did not experience mucosal healing at 4 months of treatment showed that the former had a significantly higher ADL TL than the latter (13 vs. 6.2 μg/mL) (38). Moreover, the ADL TL was significantly higher in the group that reached histological remission (17.9 μg/mL) compared to the group that did not (6.8 μg/mL). The mean value of the ADL TL at week 16 in this study was 8.13 μg/mL, with no occurrence of AAA having been observed. Furthermore, a positive correlation had been found between higher ADL TL and higher remission rate, although further research would be necessary to determine the precise level of ADL TL to be maintained.

TDM has been used to measure drug concentration levels and antibody concentrations of the anti-TNF agent in the blood. Notably, deciding whether to shorten the dosing period or switch to another drug, determining whether the effect of anti-TNF agents, such as ADL, has decreased, and identifying whether a secondary response loss, such as symptom recurrence or worsening, has occurred are all critical. In this treat-to-target era, it is also important to optimize personalized treatment. The ECCO-ESPGHAN guidelines recommends the use of early proactive TDM to guide treatment changes in patients on anti TNF therapy (54).

A recent study reported that at least 8.7 μg/mL or higher must be maintained to reach MH (38). Other adult CD studies report that maintaining a drug concentration of 5–7 and 12 μg/mL or higher is necessary to reach clinical remission and MH, respectively (56–58). Considering MH as the target in children, it would be better to maintain a drug concentration of 8 or 9 μg/mL or more during maintenance after the induction of treatment. Rinawi et al. reported that ADL TLs at 4 and 8 weeks were good predictors of combined clinical/biomarker remission at 24 weeks, and optimal ADL TL cutoff values at weeks 4 and 8 were 22.5 μg/mL and 12.5 μg/mL, respectively (59).

Although measuring and monitoring drug concentrations is helpful in improving clinical courses, opinions regarding when and at what point to test for TL and antibodies in clinical settings have varied. A recent randomized control study conducted in pediatric CD patients included a proactive drug monitoring group whose drug concentration was measured during every hospital visit at weeks 4, 8, and subsequently every 8 weeks from the beginning of treatment and a reactive group whose drug concentration was only measured when the treatment response was lost (60). Accordingly, the results of the mentioned showed that clinical remission rate at week 4 was significantly higher in the proactive than in the reactive drug monitoring group. Moreover, at weeks 48 and 72 from the start of treatment, the composite index, which was scored using three categories (clinical remission, CRP ≤0.5 mg/dL, and fecal calprotectin ≤150 μg/g), was significantly higher in the proactive than in the reactive group.

Proactive monitoring has been one of the strategies for recent treat-to-target, personalized treatment. TDM with a reliable measurement tool and objective disease activity is necessary for effective interventions tailored to each patient before irreversible intestinal damage occurs. Thus, proactive monitoring may be necessary for patients with risk factors for disease progression, including those with early disease activity of moderate or higher severity, fistulas, and advanced intestinal stenosis, as well as those who have not responded to previous IFX treatment or developed an anti-IFX antibody.

Although anti-TNF drug concentrations and antibody tests are now available, there are still cost-and-effect problems and several limitations in applying proactive drug monitoring in clinical settings. Therefore, it is necessary to determine the timing and interval of drug concentration measurements according to the treatment goals and disease characteristics of each patient and apply the appropriate ADL concentration criteria.

## Conclusion

ADL is effective in both naïve patients and those with previous experience with biologics. Beyond clinical remission, ADL has also been proven effective for MH and histological remission. The use of TDM to further enhance the effectiveness of ADL treatment is expected to reduce treatment failure and pave the way for appropriate treatment in the current treat-to-target era.

## Author Contributions

SC and BK contributed in the conception of the article, literature review, writing of the initial manuscript, and critical revision for important intellectual content. Both authors approved the final version of the manuscript and agreed to be accountable for all aspects of the work.

## Funding

This work was supported by the National Research Foundation of Korea (NRF) grant funded by the Korean government (MSIT) (No.2021M3E5D1A02015263).

## Conflict of Interest

The authors declare that the research was conducted in the absence of any commercial or financial relationships that could be construed as a potential conflict of interest.

## Publisher's Note

All claims expressed in this article are solely those of the authors and do not necessarily represent those of their affiliated organizations, or those of the publisher, the editors and the reviewers. Any product that may be evaluated in this article, or claim that may be made by its manufacturer, is not guaranteed or endorsed by the publisher.
